# Detection of Prion Protein in Urine-Derived Injectable Fertility Products by a Targeted Proteomic Approach

**DOI:** 10.1371/journal.pone.0017815

**Published:** 2011-03-23

**Authors:** Alain Van Dorsselaer, Christine Carapito, François Delalande, Christine Schaeffer-Reiss, Daniele Thierse, Hélène Diemer, Douglas S. McNair, Daniel Krewski, Neil R. Cashman

**Affiliations:** 1 Laboratoire de Spectrométrie de Masse Bio-Organique, Université de Strasbourg, IPHC, CNRS, UMR7178, Strasbourg, France; 2 Cerner Corporation, Kansas City, Missouri, United States of America; 3 McLaughlin Centre for Population Health Risk Assessment, University of Ottawa, Ontario, Canada; 4 Brain Research Centre, Department of Medicine (Neurology), University of British Columbia, Vancouver, British Columbia, Canada; Institut National de la Santé et de la Recherche Médicale, France

## Abstract

**Background:**

Iatrogenic transmission of human prion disease can occur through medical or surgical procedures, including injection of hormones such as gonadotropins extracted from cadaver pituitaries. Annually, more than 300,000 women in the United States and Canada are prescribed urine-derived gonadotropins for infertility. Although menopausal urine donors are screened for symptomatic neurological disease, incubation of Creutzfeldt-Jakob disease (CJD) is impossible to exclude by non-invasive testing. Risk of carrier status of variant CJD (vCJD), a disease associated with decades-long peripheral incubation, is estimated to be on the order of 100 per million population in the United Kingdom. Studies showing infectious prions in the urine of experimental animals with and without renal disease suggest that prions could be present in asymptomatic urine donors. Several human fertility products are derived from donated urine; recently prion protein has been detected in preparations of human menopausal gonadotropin (hMG).

**Methodology/Principal Findings:**

Using a classical proteomic approach, 33 and 34 non-gonadotropin proteins were identified in urinary human chorionic gonadotropin (u-hCG) and highly-purified urinary human menopausal gonadotropin (hMG-HP) products, respectively. Prion protein was identified as a major contaminant in u-hCG preparations for the first time. An advanced prion protein targeted proteomic approach was subsequently used to conduct a survey of gonadotropin products; this approach detected human prion protein peptides in urine-derived injectable fertility products containing hCG, hMG and hMG-HP, but not in recombinant products.

**Conclusions/Significance:**

The presence of protease-sensitive prion protein in urinary-derived injectable fertility products containing hCG, hMG, and hMG-HP suggests that prions may co-purify in these products. Intramuscular injection is a relatively efficient route of transmission of human prion disease, and young women exposed to prions can be expected to survive an incubation period associated with a minimal inoculum. The risks of urine-derived fertility products could now outweigh their benefits, particularly considering the availability of recombinant products.

## Introduction

Transmissible spongiform encephalopathies (TSEs), or prion diseases, such as scrapie, bovine spongiform encephalopathy (BSE) and Creutzfeldt-Jakob disease (CJD), are sub-acute neurodegenerative disorders in which the infectious agent is an aggregated, misfolded host prion protein that propagates through ‘template directed misfolding’. The disease-associated protein isoform, generically designated PrP^TSE^, is derived from a normal, non-infectious, host-derived cellular isoform termed PrP^C^. The most common human TSE, CJD, occurs in forms which are sporadic (sCJD), iatrogenic (iCJD) or genetic (e.g. familial CJD: fCJD). The emergence of a new variant CJD (vCJD) in the UK and France, almost certainly the result of human consumption of BSE-contaminated bovine tissues, has affected 216 individuals in 11 countries worldwide as of March 2010 [1]. Although the primary zoonotic epidemic of vCJD is probably in decline, a survey of lymphoreticular tissues in the UK estimated that 237 people per million population (95% confidence interval: 49–692 per million) were ‘silent carriers’ of vCJD [2]. These results suggest that many countries may have asymptomatic citizens 'incubating' the disease for decades. Thus, it is possible that secondary outbreaks may occur through iatrogenic transmission via organ transplantation, contaminated surgical instruments, and human-derived therapeutic products; indeed, four cases of blood-borne transmission of the vCJD prion agent have already been documented in the UK. Iatrogenic transmission of classical CJD has also been demonstrated through dural and corneal transplantation and neurosurgical instruments. Notably, in the 1990s 194 individuals were infected through intramuscular injections of pituitary growth hormone derived from human cadavers [3]. The latter episode not only demonstrated the high infectivity of parenterally injected CJD prions (recipient disease rates exceeded 10% in some French batches), but also raised the possibility that chromatography systems used to purify therapeutic hormones and peptides may enrich human prions. Four cases of iatrogenic transmission of CJD through fertility treatment involving human pituitary-derived gonadotropin have also been reported in Australia [4].

It is now clear that infectious prions are excreted into the urine in renal disease states. Studies from the Aguzzi laboratory in 2005 convincingly demonstrated the presence of fully infectious prion particles in the urine of scrapie mice with co-existing renal inflammation [5]. Although PrP^TSE^ levels were below the limits of detection by immunoblotting in this study, intracerebral inoculation of concentrated urine transmitted scrapie in host mice. More recent work has demonstrated that renal pathology is not required for urinary prion excretion in experimental animals. In 2006, the Gabizon group [6] first demonstrated prionuria in the absence of renal inflammation by inducing prion disease in naïve hamsters through inoculation of scrapie urine. In 2007, Gregori and colleagues [7] and Murayama and colleagues [8] demonstrated detectable levels of PrP^TSE^ in the urine of scrapie hamsters for the first time. Urinary PrP^TSE^ was only detectable at the symptomatic stage of the disease. However, these findings do not imply that urine is ‘safe’ if PrP^TSE^ is undetectable; Aguzzi and colleagues failed to detect PrP^TSE^ in urine subsequently proven to be infectious [5]. Furthermore, the studies of both Aguzzi [5] and Gabizon [6] found infectivity in the urine during the presymptomatic incubation period before the appearance of clinical disease. Gonzalez-Romero and colleagues recently confirmed the presence of templating-competent PrP^TSE^ in the urine of experimentally infected animals using protein misfolding cyclic amplification [9]. Using a rigorous quantitative method, Gregori and colleagues [7] have recently reported that the titer of prions in urine of scrapie-infected hamsters was several units of infectivity per mL, similar to that observed in hamster blood.

Collectively, these studies suggest that the urine of prion-infected individuals may be infectious before the appearance of clinical signs – even before PrP^TSE^ is detectable in the urine. These and other studies [10], [11] also indicate that, while renal disease may facilitate prionuria, such pathology is not a prerequisite for urinary prion transmission. Recently, Kuwabara and colleagues [12] have identified prion protein in urine-derived gonadotropins. Although this class of products is not administered to patients for extended periods, the number of injections and the amount of total protein administered could expose patients to a potential risk of prion transmission. Using advanced proteomics methods, we now report the detection of prion protein in commercially available urine-derived gonadotropins, but not in recombinant fertility products.

## Results

### Identification of non-gonadotropin proteins in human chorionic gonadotropin (hCG) preparations using electrophoresis and mass spectrometry (MS)

**Figure 1:**
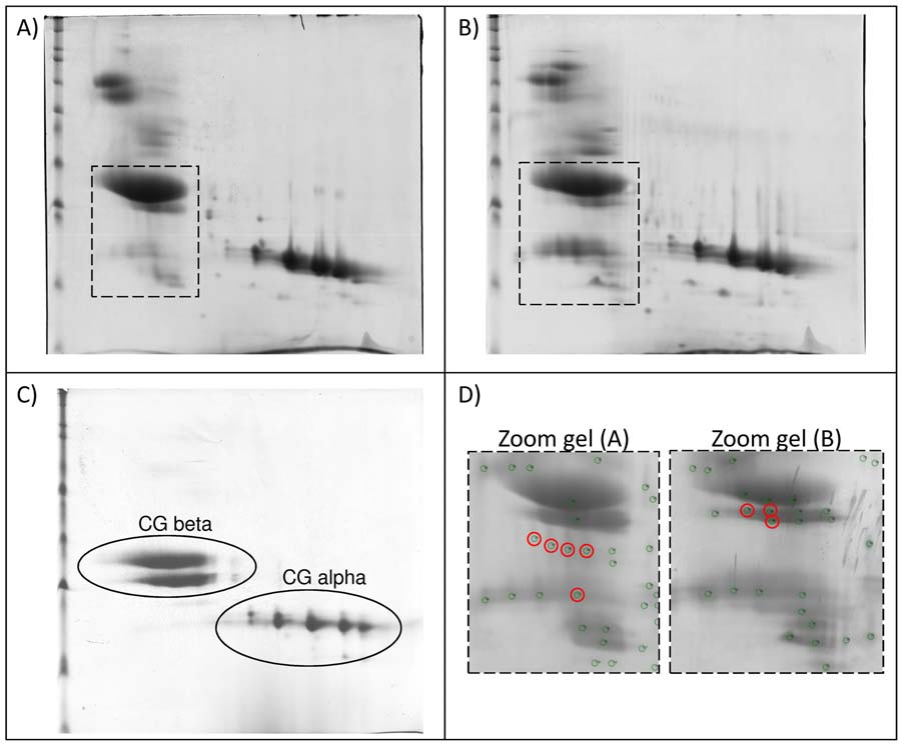
2D gel electrophoresis separation of urinary and recombinant hCG products (6,500 IU loaded). (A) u-hCG manufacturer A. (B) u-hCG Manufacturer B. (C) r-hCG Manufacturer D. (D) Zoom views on the regions of gels (A) and (B) with spots containing PrP peptides encircled.

After 2D gel electrophoresis of hCG products (Figure 1), protein spots found to be present in the molecular weight range of approximately 14–70 kDa in u-hCG and recombinant-hCG (r-hCG) were analyzed by electrospray ionization MS (ESI–MS/MS), and identified using internationally available databases.

Several non-gonadotropin proteins were identified in the u-hCG derived preparations (Table 1) but not in the r-hCG preparation. For u-hCG-manufacturer A, prion protein was identified as one of the major non-gonadotropin proteins present. Within urinary impurities, major prion protein (Swissprot accession number P04156) was identified in several 2D gels as a series of spots in the mass range 21–25 kDa for u-hCG-manufacturer A and at about 29 kDa for u-hCG-manufacturer B. The isoelectric point (pI) ranged from four to five for both samples. The fact that major prion protein was identified in several spots is probably due to heterogeneous glycosylation and degraded prion protein forms [13]. In contrast, no prion protein was identified in the r-hCG preparation.

**Table 1:** Non-gonadotropin protein profile identified in u-hCG products.

N	Identified human proteins	Accession number	u-hCG manufacturer A	u-hCG manufacturer B
1	Major prion protein	P04156	X	X
2	Apolipoprotein D	P05090	X	X
3	Beta-2-glycoprotein 1	P02749	X	X
4	Complement decay-accelerating factor	P08174	X	X
5	Alpha-1-microglobulin (protein AMBP)	P02760	X	X
6	CD59 glycoprotein	P13987	X	X
7	Uromodulin	P07911	X	X
8	Growth/differentiation factor 15	Q99988	X	X
9	Endosialin	Q9HCU0	X	X
10	Protein delta homolog 1	P80370	X	X
11	Fibrillin-1	P35555	X	X
12	Matrix metalloproteinase-9	P14780	X	X
13	Plasminogen	P00747	X	
14	Complement factor B	P00751	X	
15	Tumor necrosis factor receptor superfamily member 1B	P20333	X	
16	Hepatocyte growth factor activator	Q04756	X	
17	EGF-containing fibulin-like extracellular matrix protein 1	Q12805	X	
18	Tumor necrosis factor receptor superfamily member 1A	P19438	X	
19	Connective tissue growth factor	O18739	X	
20	Basement membrane-specific heparan sulfate proteoglycan core protein	P98160	X	
21	Plasma serine protease inhibitor (protein C inhibitor)	P05154		X
22	Insulin-like growth factor-binding protein 7	Q16270		X
23	Zinc-alpha-2-glycoprotein	P25311		X
24	Folate receptor alpha	P15328		X
25	Kininogen-1	P01042		X
26	Protein S100-A7 (Psoriasin)	P31151		X
27	Pro-epidermal growth factor	P01133		X
28	Fibulin-2	P98095		X
29	Collagen alpha-1(III) chain	P02461		X
30	Apolipoprotein(a)	P08519		X
31	Probable carboxypeptidase PM20D1	Q6GTS8		X
32	Complement factor H-related protein 1	Q03591		X
33	Cystatin-M	Q15828		X

u-hCG  =  urinary human chorionic gonadotropin.

**Figure 2:**
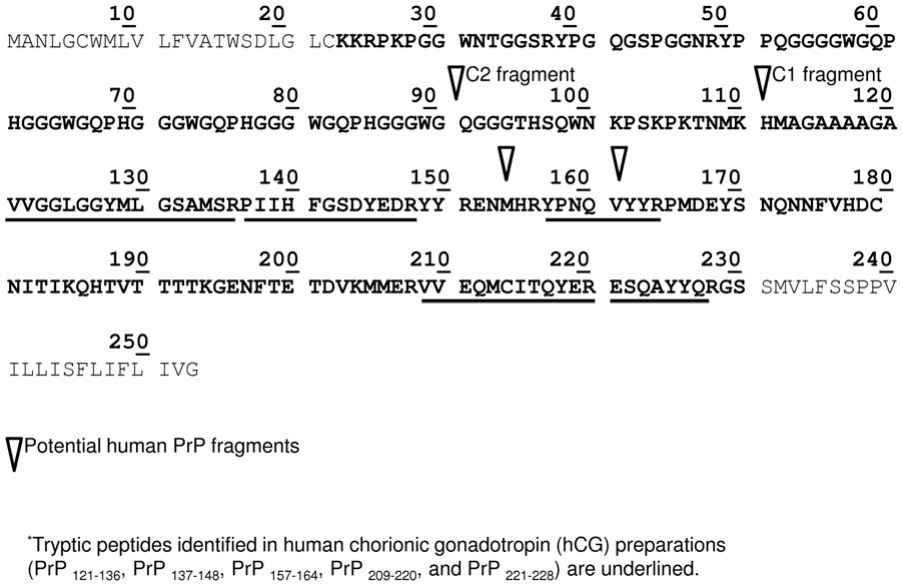
Human prion protein (PrP) sequence (mature form in bold). ^*^Tryptic peptides identified in human chorionic gonadotropin (hCG) preparations (PrP _121-136_, PrP _137-148_, PrP _157-164_, PrP _209-220_, and PrP _221-228_) are underlined.

Considering the importance of these findings, liquid chromatography tandem MS (LC-MS/MS) was conducted to confirm the presence of prion protein in both u-hCG products. The tryptic peptides identified by LC-MS/MS in u-hCG preparations are reported in Figure 2. A total of five peptides were identified from the mature human prion protein sequence covering amino acids 121–228 which is in agreement with recently published characterization of the truncated prion protein forms in human urine [14]. MS/MS spectra of all the peptides identified are provided in Figure S1.

### Separation of gonadotropin and non-gonadotropin proteins in human menopausal gonadotropin (hMG) using electrophoresis

**Figure 3:**
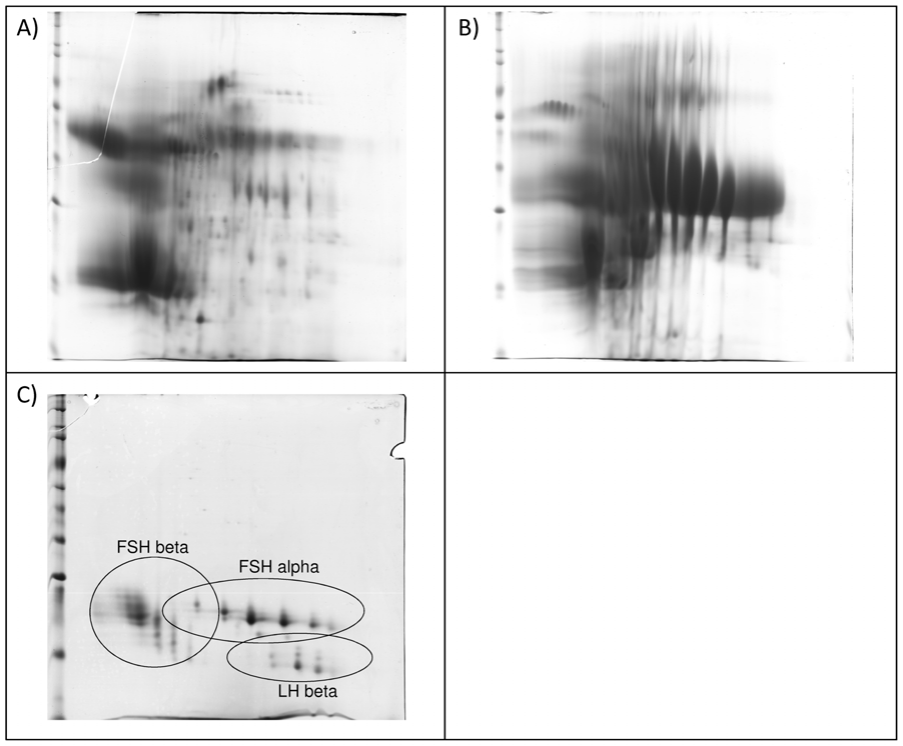
2D gel electrophoresis separation of urinary hMG and recombinant hFSH/hLH products. (A) hMG Manufacturer A (600 IU loaded). (B) hMG Manufacturer C (1,125 IU loaded). (C) r-hFSH/r-hLH Manufacturer D (1,125 IU loaded).

Two samples of hMG and one of a combination of recombinant human follicle-stimulating hormone and recombinant human luteinizing hormone (r-hFSH/r-hLH) were analyzed by 2D gel electrophoresis (Figure 3). According to the high amount of impurities known to be present in hMG (manufacturer A), a lower amount (600 IU FSH) of this sample was loaded. Since no information was available on the purity of hMG (manufacturer C), it was handled as hMG-HP preparation by loading a total of 1,125 IU FSH. Comparative analysis indicated that both hMGs tested were less pure than hMG-HP and contained high levels of total proteins, mainly urinary impurities.

### Identification of non-gonadotropin proteins in hMG-HP preparations using electrophoresis and MS

**Figure 4:**
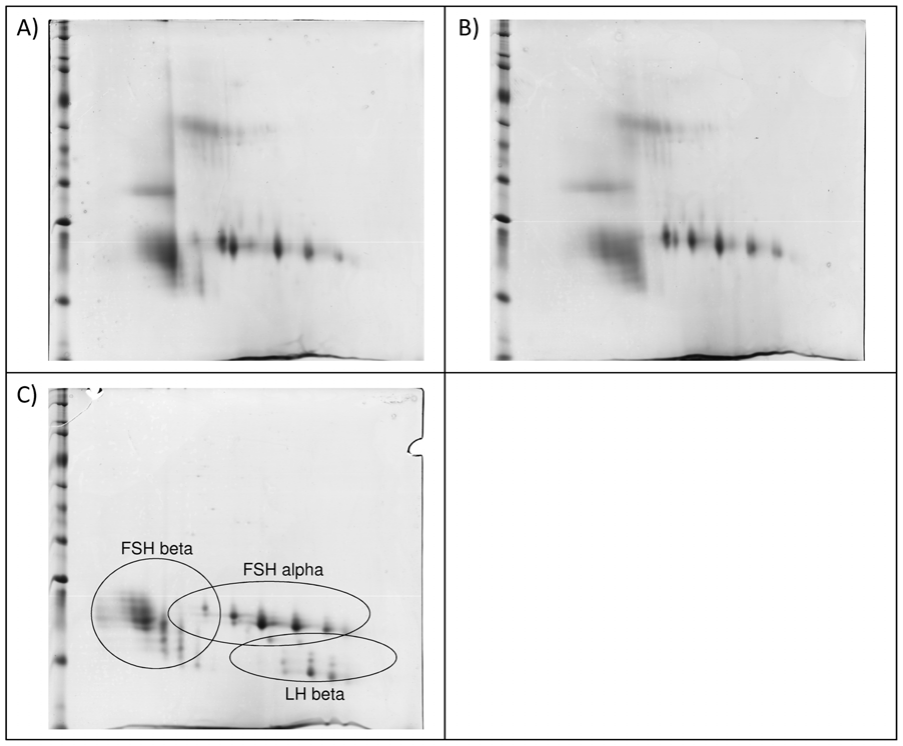
2D gel electrophoresis separation of highly purified urinary hMG and recombinant hFSH/hLH products (1,125 IU loaded). (A) hMG-HP Manufacturer A-Product 1. (B) hMG-HP Manufacturer A-Product 2. (C) r-hFSH/r-hLH Manufacturer D.

After 2D gel electrophoresis of two batches of hMG-HP (Figures 4A and 4B), protein spots were analyzed by LC-MS/MS, and identified using internationally available databases.

In total, 34 non-gonadotropin proteins were identified in the protein profile of the hMG-HP preparations (Table 2). No impurities were identified in the r-hFSH/r-hLH preparation (Figure 4C). Considering previous proteomics studies [15], the new impurities identified in hMG-HP products by manufacturer A increased the number of non-gonadotropin proteins identified by the scientific community to 39.

**Table 2:** Non-gonadotropin protein profile identified in hMG-HP products.

N	Identified impurities	Accession number	hMG-HP manufacturer A Product 1	hMG-HP manufacturer A Product 2
1	Plasma serine protease inhibitor (protein C inhibitor)	P05154	XX	XX
2	Apolipoprotein D	P05090	XX	XX
3	Insulin-like growth factor-binding protein 7	Q16270	XX	XX
4	Leukocyte elastase inhibitor	P30740	XX	XX
5	Alpha-2-antiplasmin	P08697	XX	X
6	Zinc-alpha-2-glycoprotein	P25311	X	XX
7	Serum albumin	P02768	X	X
8	Folate receptor alpha	P15328	X	X
9	Urokinase plasminogen activator surface receptor	Q03405	X	X
10	Afamin	P43652	X	X
11	Kininogen-1	P01042	X	X
12	Reversion-inducing cysteine-rich protein with Kazal motifs	O95980	X	X
13	Alpha-1-antitrypsin	P01009	X	X
14	Plasminogen	P00747	X	X
15	Beta-2-glycoprotein 1	P02749	X	X
16	Thrombospondin type-1 domain-containing protein 4	Q6ZMP0	X	X
17	Protein S100-A7 (Psoriasin)	P31151	X	X
18	Alpha-1-acid glycoprotein 1	P02763	X	X
19	Complement decay-accelerating factor	P08174	X	X
20	Sialate O-acetylesterase	Q9HAT2	X	
21	Alpha-1-microglobulin (protein AMBP)	P02760	X	
22	Ig kappa chain C region	P01834	X	
23	Ig gamma chain C region	P01857	X	
24	Peptidoglycan recognition protein	O75594	X	
25	Golgi phosphoprotein 2	Q8NBJ4		X
26	Delta-like protein 1	O00548		X
27	Death-associated protein kinase 1	P53355		X
28	CD27 antigen	P26842		X
29	Serotransferrin	P02787		X
30	TNF receptor superfamily member 21	O75509		X
31	CD59 glycoprotein	P13987		X
32	Folate receptor gamma	P41439		X
33	Platelet endothelial aggregation receptor 1	Q5VY43		X
34	TNF receptor superfamily member 4	P43489		X

hMG-HP  =  highly purified urinary human menopausal gonadotropin.

### Targeted prion protein quantification in gonadotropin preparations using liquid chromatography selected reaction monitoring (LC-SRM)

LC-SRM analysis was performed directly on all hCG samples to allow specific detection of tryptic peptides from the prion protein, confirming unambiguously the 2D gel data from urine-derived hCG samples. The same technique was used to test the other products included in this study (u-hCG, hMG and hMG-HP), and was able to detect and quantify the human prion protein (Table 3).

**Table 3:** Results of prion protein peptides analysis in gonadotropin preparations by LC-SRM.

Commercial product	Manufacturer	Concentration	Prion detection (peptide 209-220, peptide 221-228)	Prion quantification (peptide 209-220)		
				Prion content (pmoles/vial)	CV (%)	Limit of detection (pmoles/vial)
u-hCG	A	5,000 IU	Yes	116.21	5	2.88
	B – product 1	5,000 IU	Yes	30.38	7	2.00
	B – product 2	5,000 IU	Yes	15.78	6	0.62
hMG	A	75 IU	Yes	6.78	6	1.35
	C	75 IU	Yes	5.38	12	0.96
hMG-HP	A – product 1*	75 IU	Yes	0.21	10	0.01
	A – product 2*	75 IU	Yes	0.10	13	0.01
r-hCG	D	250 µg	No	<LOD	-	0.47
r-hFSH/r-hLH	D	150 IU/75 IU	No	<LOD	-	0.09
r-hLH	D*	75 IU	No	<LOD	-	0.04

For all products a single vial was used for measurements. For products with *, six vials were pooled in order to minimize protein material losses.

LC-SRM  =  liquid chromatography selected reaction monitoring; CV  =  coefficient of variation; u-hCG  =  urinary human chorionic gonadotropin; hMG  =  urinary human menopausal gonadotropin; hMG-HP  =  highly purified hMG; r-hCG  =  recombinant hCG; r-hFSH  =  recombinant human follicle-stimulating hormone; r-hLH  =  recombinant human luteinizing hormone.

It should be noted that primary experiments were performed using only one vial of each product. Among the urinary preparations, hMG-HP showed the presence of prion protein but the level was not quantifiable (data not shown). The experiment was then repeated with 6 pooled vials and prion presence was confirmed. No trace of prion protein was detected when the same process (using a larger number of vials) was carried out for the recombinant preparation r-hLH (Table 3).

The tryptic prion peptides PrP (209–220) and PrP (221–228) were used for SRM detection and peptide PrP (209–220) for quantification. As the human PrP peptide sequence (221–228) is identical to the related bovine PrP peptide, it was used to simultaneously evaluate the presence of human and bovine prion sequence. These tryptic prion peptides are located in the human prion carboxy-terminal sequence, and thus allow the detection of several potential truncated prion protein forms (see Figure 2) [13], [16]. LC-SRM is therefore suitable to evaluate the potential presence of bovine prion peptides in recombinant products, if bovine prion protein is present at levels higher than the limit of detection (LOD). The LOD was experimentally established and found to be product-dependent. According to the results reported in Table 3, no human or bovine prion peptides were detected in r-hCG, r-hLH, and r-hFSH/r-hLH products from manufacturer D.

## Discussion

Using classical proteomic analyses, prion protein was detected for the first time in two u-hCG preparations, and was among 33 different non-gonadotropin proteins identified as contaminants of these pharmaceutical products. In contrast, r-hCG preparations were negative for prion proteins.

In one of the two u-hCG products tested, human prion protein was among the ten major contaminants. The fact that prion protein sequences were identified in several spots on our 2D electrophoresis gels is likely due to the presence of heterogeneous glycosylation and degraded prion protein forms. It is also worth noting that in the u-hCG preparation of manufacturer A, plasminogen was identified among the urinary impurities; plasminogen has been identified as a binding protein for disease-associated prion protein [17].

Both hMG and hMG-HP were tested using 2D gel electrophoresis. The results confirmed that the two hMGs tested were less pure than hMG-HP and contained a large number of total proteins (mainly represented by urinary impurities). Non-gonadotropin proteins present in hMG-HP products were identified by MS, resulting in 34 co-purified contaminants. Only gonadotropin proteins were seen in all the recombinant preparations analyzed by 2D gel electrophoresis. Using this 2D-gel/LC-MS/MS proteomic workflow, prion proteins were identified only in u-hCG and not in hMG-HP preparations. In parallel, a targeted proteomic approach (LC-SRM) was developed to detect human prion proteins which are sensitive to proteases. The method was optimized to provide quantitative data in each container of product. This is the most sensitive MS-based quantification technique currently available with a limit of detection in the low femtomolar range. This approach for prion protein detection, identification and quantification was used on all gonadotropin pharmaceutical preparations included in our study, including both urinary (hCG, hMG, hMG-HP) and recombinant products.

All urine-derived preparations tested, produced by different manufacturers, showed the presence of human prion proteins in varying amounts. These findings demonstrate that the purification processes for different urine-derived preparations are unable to remove prion proteins from the source material and that the process controls employed do not permit the identification of this contaminant.

Do the prion protein peptides detected in this study originate from infectious prions? Preparation of tryptic peptides is preceded by solubilization in 8M urea, which is adequate to disaggregate and denature the disease-associated isoform of the prion protein rendering it susceptible to trypsin digestion. It is also clear that native and diseased isoforms of the prion protein share affinity for chromatography substrates utilized to purify peptide hormones [3]. Finally, infectious prions can range down in size to oligomers of a few dozen prion protein molecules [18], which would be undetectable by existing biochemical methodologies including MS methods employed in this study.

Although no cases of human prion disease due to the use of urinary gonadotropins have been recognized to date, the epidemiological signal for transmission may be difficult to detect. Each year, more than 300,000 young women in the US and Canada are prescribed urine-derived gonadotropins for infertility. Although the Food and Drug Administration and Health Canada once considered these products to be in the lowest category of risk for prion disease transmission, the discovery of full infectivity in the urine of nephritic scrapie-infected mice in 2005 led to new requirements for product labeling and a review of donor procedures and manufacturing processes. Additional recent findings suggest that urinary prion excretion can occur without renal pathology [6], [7]. These results warrant a reassessment as to whether the risks of urine-derived fertility products could now outweigh their benefits, particularly considering the availability of recombinant products that do not require human urine as a substrate.

Although urinary gonadotropins have been previously characterized as safe [19], [20], this opinion may be overly optimistic in view of the present findings, supported by results from other recently published studies. Notably, blood products were once also considered ‘safe’, based on the lack of detectable prions in vCJD using an inadequately sensitive mouse bioassay [21]. In line with recent published studies, the 2010 updated World Health Organization tables on ‘*Tissue infectivity distribution in transmissible spongiform encephalopathy’* moved urine from the category of *‘Tissues with no detectable infectivity’* to the category of *‘Lower-infectivity tissue’* (the latter category includes blood) [22].

Current urine collection systems pool the urine of thousands of donors and, unlike the blood collection system, do not allow for donor tracing. There is also no mechanism of ensuring that the designated donor is actually the one who provides the urine, as donation is normally done at home. However, even if donor management and tracing were flawless, the fact that prionuria may exist well before the onset of clinically overt prion disease, without being detectable by current methods, remains a cause for concern. Furthermore, the now indisputable detection of prions in urine of experimental animals, the lack of a species barrier for human-to-human transmission, the relative efficiency of the intramuscular injection route for prion transmission, and the young age of fertility drug recipients all support application of the ‘precautionary principle’ for urinary derived pharmaceuticals. As risk management paradigms shift towards more proactive approaches intended to ‘anticipate and prevent’ emerging risks [23]–[26], a careful examination of the risk of transmission of human prion disease through the use of urine-derived hormones and peptides would appear to be warranted.

## Materials and Methods

### Source of material

Commercial products of urinary human chorionic gonadotropin (u-hCG) from Ferring (Switzerland) and Organon (Denmark), human menopausal gonadotropin derived from urine (hMG) from Ferring (Switzerland) and Livzon (China); and hMG highly purified (hMG-HP) from Ferring (Switzerland) were used (Table 4). In addition, recombinant human chorionic gonadotropin (r-hCG), serum-free recombinant human follitropin alfa in association with lutropin alfa (r-hFSH + r-hLH) and human lutropin alfa alone (r-hLH) were all from Merck Serono (Switzerland) (Table 4).

**Table 4:** Characteristics of commercial products analyzed.

Commercial product	Manufacturer	Strength	Country producer
u-hCG	A	5000 IU	Switzerland
	B – product 1	5000 IU	Denmark
	B – product 2	5000 IU	Denmark
hMG	A	75 IU	Switzerland
	C	75 IU	China
hMG-HP	A – product 1	75 IU	Switzerland
	A – product 2	75 IU	Switzerland
r-hCG	D	250 µg	Switzerland
r-hFSH/r-hLH	D	150IU/75IU	Switzerland
r-hLH	D	75 IU	Switzerland

u-hCG  =  urinary human chorionic gonadotropin; hMG  =  urinary human menopausal gonadotropin; hMG-HP  =  highly purified hMG; r-hCG  =  recombinant hCG; r-hFSH  =  recombinant human follicle-stimulating hormone; r-hLH  =  recombinant human luteinizing hormone.

### Identification of non-gonadotropin proteins using electrophoresis and MS

#### a) Sample preparation

A total amount of 6,500 IU of hCG samples, 600 IU of hMG sample A and 1,125 IU of hMG sample C, 1,125 IU of hMG-HP samples and 1,125 IU r-hFSH/r-hLH sample were pretreated by ultrafiltration to remove non-protein product excipients such as lactose or mannitol which are incompatible with the first dimension of 2D SDS-PAGE (VIVASPIN-2 filter, Sartorius VS0291; cut off: 3,000 Da, centrifugation at 4,000 g, temperature was constantly maintained at 4°C). The volume of solution was reduced to about 0.6 mL of pure water.

#### b) SDS-PAGE

Samples were loaded onto a 2D SDS–PAGE gel. Proteins were separated on a first dimension according to their pI at 86,000 VH, and then on a second dimension according to their molecular weight. Following 2D separation, the gels were stained by silver or Coomassie blue staining. Detected proteins were removed from the gel using a gel cutter (PROTEINEERsp, Bruker Daltonics, Bremen, Germany), washed, reduced, alkylated and dehydrated automatically with a MassPrep robot (Waters, Milford, MA). The gel pieces were digested with porcine trypsin (Promega, Madison, WI, USA). The resulting tryptic peptides were extracted with 60% acetonitrile in 0.5% formic acid and analyzed by nanoflow liquid chromatography tandem mass spectrometry (nanoLC-MS/MS).

#### c) Protein identification by NanoLC-MS/MS analysis

NanoLC-MS/MS analysis was performed using an Agilent 1100 series nanoLC-Chip system (Agilent Technologies, Palo Alto, USA) coupled to an HCT Plus ion trap (Bruker Daltonics, Bremen, Germany). The chip was composed of a Zorbax 300SB-C18 analytical column (43 mm ×75 µm, with a 5 µm particle size) and a Zorbax 300SB-C18 enrichment column (40 nL, 5 µm). Elution of the peptides was performed at a flow rate of 300 nL.minute^−1^ with a 8–40% linear gradient (solvent B, 98%ACN/0.1%FA) over 7 minutes.

The voltage applied to the capillary cap was optimized to −1,750 V and the mass range was 250–2,500 *m*/*z*. For tandem MS experiments, the system was operated in data-dependent acquisition mode with automatic switching between MS and MS/MS. The three most abundant precursor ions were selected to be further isolated and fragmented. The MS/MS scanning was performed in the ultrascan resolution mode at a scan rate of 26.000 *m*/*z* per second. A total of six scans were averaged to obtain an MS/MS spectrum.

Mass data collected during nanoLC- MS/MS analysis were processed, converted into *.mgf files and were interpreted using a local Mascot server (Matrix Science, London, UK). Searches were performed against the SwissProt database (57.4 version, 470,369 entries) using a target/decoy approach and without molecular weight or isoelectric point restrictions. The tolerance on mass measurements was set to 0.20 Da for both MS and MS/MS modes. One tryptic missed cleavage per peptide was allowed and some variable modifications were taken into account such as carbamidomethylation for cysteine and oxidation for methionine. Validation criteria were applied in order to retain no decoy hit.

### Targeted prion protein quantification in gonadotropin preparations using LC-SRM

#### a) Sample preparation

After removal of the non-protein product excipients as described for the 2D gel electrophoresis, the total protein content was determined by Bradford assay using the Bio-Rad Protein Assay Kit (Bio-Rad Laboratories, Hercules, CA, USA) according to the manufacturer's instructions for the Microplate Protocol, with a working range of 1–25 µg. Bovine serum albumin was used as standard. Samples were evaporated to dryness and rediluted with 200 µL of 8 M urea, 0.1 M NH_4_HCO_3_. Reduction was performed with addition of 3.5 µl of 700 mM DTT, 37°C, 30 minutes (final concentration 12 mM) and alkylation with addition of 12 µl of 700 mM IAA, 25°C, dark, 1 hour (final concentration 40 mM). In order to perform the tryptic digestion, this solution was diluted with 1,500 µL of 0.1 M fresh NH_4_HCO_3_. Digestion was carried out by adding 1 µg of bovine trypsin (Promega) per 100 µg of proteins and maintained at 37°C overnight. Desalting and further concentration was carried out by adsorption using C18 Sep-Pak cartridges (Sep-Pak Vac 1cc 50 mg, tC18, Waters) and peptides were eluted with 600 µL of 50%ACN/0.1%FA. After evaporation to dryness, the peptide fraction was re-dissolved in water to a final concentration of 1 µg/µL.

#### b) LC-SRM quantification of human PrP

The approach developed was mainly based on the AQUA strategy previously described by Gerber et al. for the absolute quantitative analysis of proteins [27].

Stable isotope labeled internal standard peptides (Sigma Aldrich, St. Louis, MO) were introduced in protein samples just before proteolytic digestion. Both the isotope labeled AQUA peptide and the unlabeled native peptide (produced during proteolytic cleavage of the target protein) were then measured by LC-SRM. Similar to small molecule isotope dilution strategies, the isotope-labeled standard and the analyte are identical except for their masses. In an analogous fashion to classic isotope dilution approaches, the mass difference allows the mass spectrometer to differentiate between the two nearly identical species. Since the amount of internal standard is known, and the ratio between amounts of internal standard and analyte can be determined from the mass spectra, the amount of the analyte peptide can be calculated.

The quantification was performed directly on vials or pooled vials. Peptides already detected and identified in 2D gel spots by the classical LC-MS/MS proteomic approach were targeted by LC-SRM allowing a specific detection of the four peptide sequences of the human PrP: 137–148, 157–164, 209–220, and 221–228. A fifth peptide, corresponding to peptide 209–220 with the methionine oxidized, was also targeted. Three different amounts of standard peptides were spiked in (250, 750, and 2,250 femtomoles) in order to make sure that the final quantification was performed in the linear range. All analyses were performed in triplicate to evaluate the coefficient of variance. Results were given in picomoles per vial.

Because of the low amounts of standard peptides spiked in the samples, special care had to be taken to prepare these standard solutions. To minimize expected losses of peptides by irreversible adsorption on the walls of the tubes at very low concentrations, dilution of the stock solution was made in low-binding 500 µL tubes. The dilutions from these stock solutions were made extemporaneously, briefly mixed and spiked in samples within less than 2 min after dilution. We have verified that at the final diluted concentrations, there is no signal intensity loss when 1 µL is analyzed in less than 3 min. The following dilution process has been used: 1 nanomole of lyophilized AQUA peptides was dissolved in 20 µl of 10%FA, sonicated for 3 min and then 180 µl of 0.1%FA was added and vortexed to obtain a stock solution at 5 pmol/µl.

#### c) Micro LC separation

Separation was made on an Agilent 1100 HPLC system equipped with a C18 column (Zorbax 300 SB C18 Agilent, 150×0.3 mm, 3.5 µm) using a water/acetonitrile gradient at 4 µL.minute^−1^ (2–22% acetonitrile in 17 minutes) and a preconcentration system (Zorbax SB C18 Agilent, 17×1 mm, 3.5 µm, reverse mode).

#### d) SRM method setup

A triple quadrupole mass spectrometer (QQQ G 6410, Agilent Technologies) was used. The best transitions per peptide were optimized using the Peptide Optimizer software tool. One fully tryptic peptide (209–220) was used for quantification. Peptide (221–228) gave in all samples in which PrP was detected a lower amount than peptide (209–220) indicating that the PrP must undergo C-terminal cleavages. Hence, the quantification using the more internal peptide (209–220) was more relevant. The other two peptides (137–148 and 157–164) were excluded for quantification as they correspond to proline cleavages and displayed incomplete and irreproducible trypsin cleavages. The list of the optimized transitions is given in Table 5 for the native peptides and the internal isotope labeled standard peptides. Dynamic SRM method setup was used with a dwell time of 200 ms per transition. The most intense fragment was used as a quantifier and the two other as qualifiers; the tolerances for the fragments' ratios are listed in Table 5.

**Table 5:** Optimized transitions for prion protein native peptides and isotope labeled standard peptides.

Peptides	Precursor ion m/z (charge state)	Quantifier (A) m/z (charge state, fragment type)	Qualifier 1 (B) m/z (charge state, fragment type)	Qualifier 2 (C) m/z (charge state, fragment type)	B/A	C/A
Native 209-220	778,4 (2+)	696,4 (1+, y5)	1228,6 (1+, y9)	1100,5 (1+, y8)	0,35+/−25%	1+/−25%
AQUA 209-220	783,4 (2+)	706,4 (1+, y5)	1238,6 (1+, y9)	1110,6 (1+, y8)	0,35+/−25%	1+/−25%
Native 221-228	522,7 (2+)	629,3 (1+, y4)	466,3 (1+, y3)	700,3 (1+, y5)	1+/−30%	1+/−30%
AQUA 221-228	527,8 (2+)	639,4 (1+, y4)	476,3 (1+, y3)	710,4 (1+, y5)	1+/−30%	1+/−30%

SRM was used to follow peptide fragments, the most intense fragment was used as a quantifier (A) and the two secondary fragments were used as qualifiers (B and C).

## Supporting Information

Figure S1 **MS/MS spectra of peptides identified in the 2-D gel electrophoresis spots as PrP tryptic peptides.**
(PDF)
