# Supplementary material for: Detection of Prion Protein in Urine-Derived Injectable Fertility Products by a Targeted Proteomic Approach
Source: PLoS One. 2011 Mar 23;6(3):e17815. doi: 10.1371/journal.pone.0017815 (PMC3063168; doi:10.1371/journal.pone.0017815)
Supplement: Figure S1 — MS/MS spectra of peptides identified in the 2-D gel electrophoresis spots as PrP tryptic peptides. (PDF) [file pone.0017815.s001.pdf]

Figure S1

MS/MS spectra of peptides identified in the 2-D gel electrophoresis spots as PrP tryptic peptides

Sequences identified using the Mascot search engine:

209-220: VVEQMCITQYER

209-220: VVEQMoxCITQYER

137-148: PIIHFGSDYEDR

157-164: YPNQVYYR

221-228: ESQAYYQR

121-136: VVGGLGGYMLGSAMoxSR

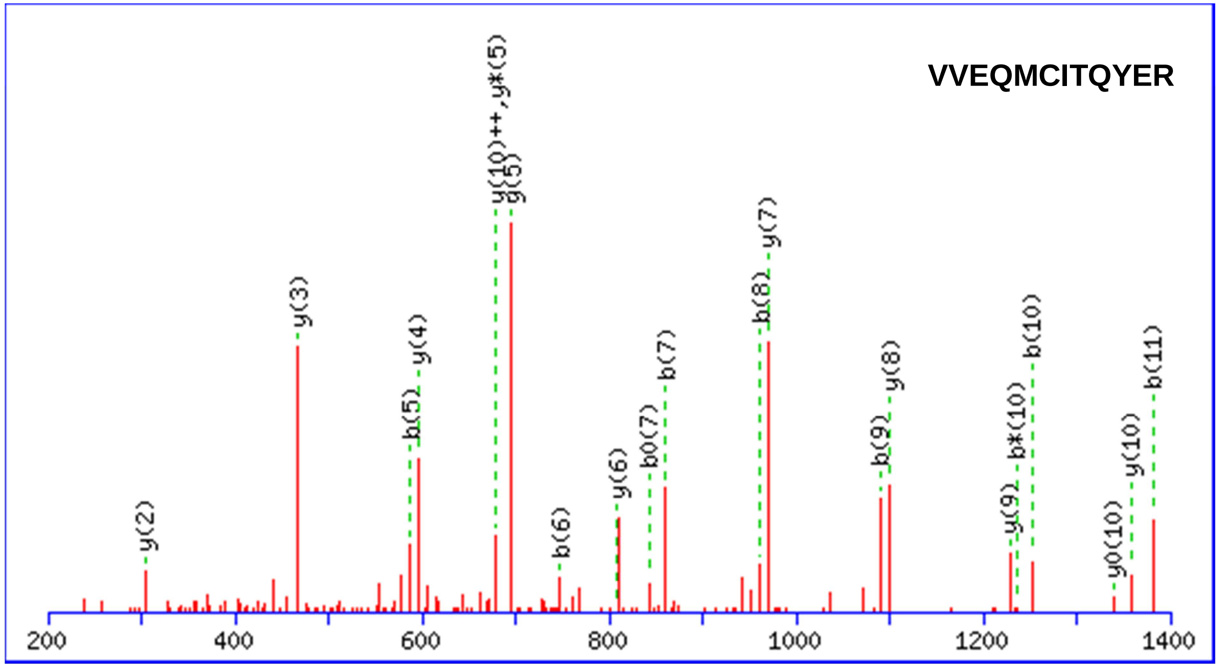

M=1554.8 Da. MS/MS from 778.4 (2+)

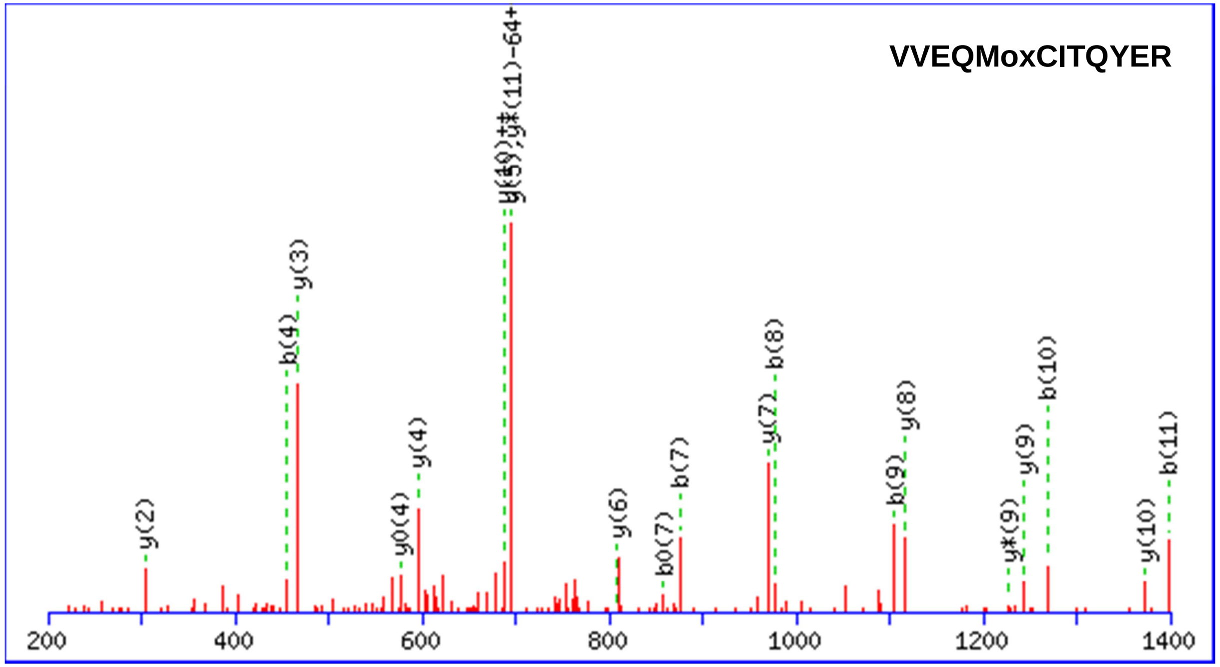

M=1570.7 Da. MS/MS from 786.4 (2+)

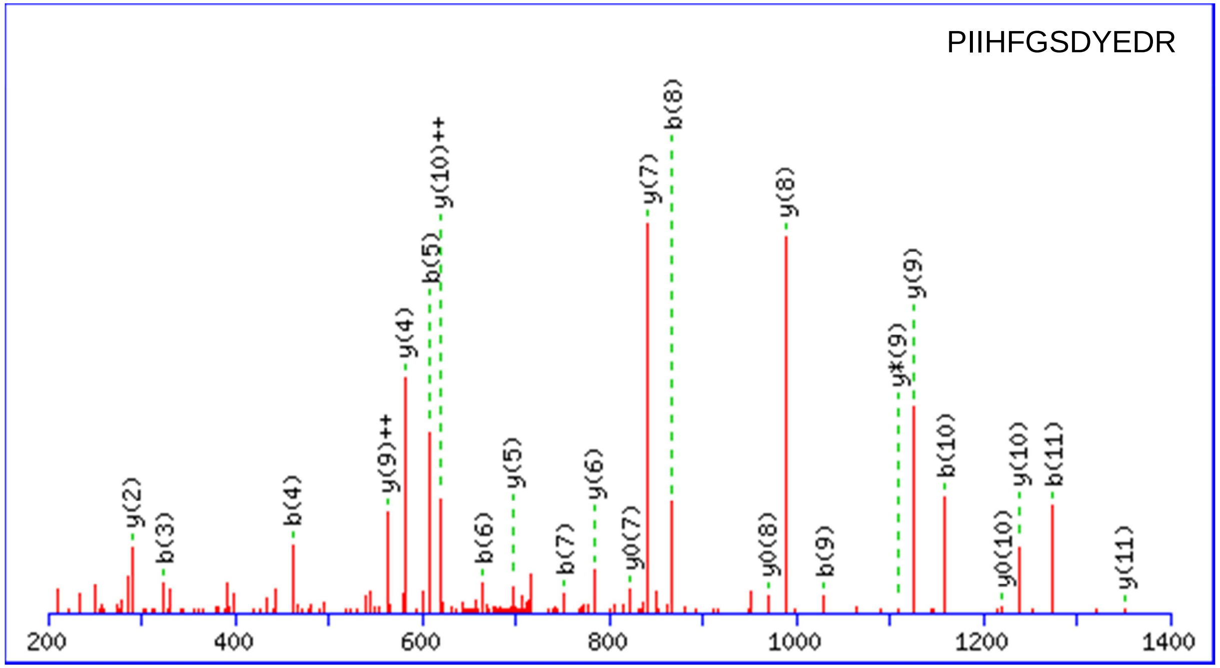

M=1447.7 Da. MS/MS from 724.8 (2+)

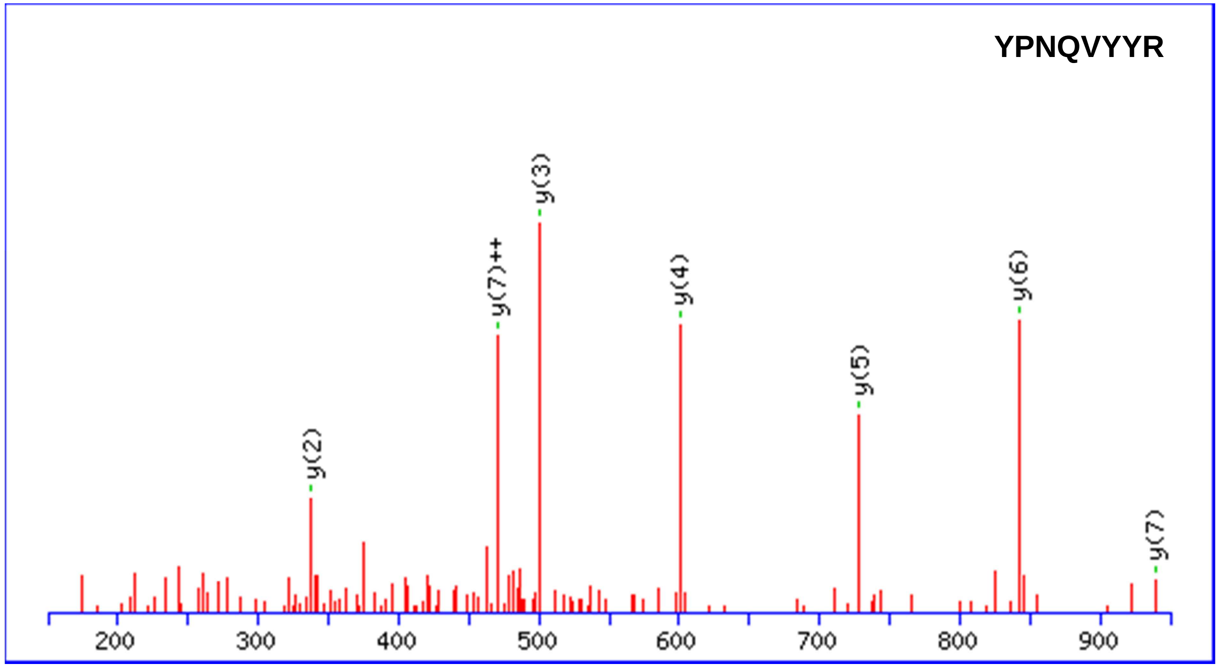

M=1101.5 Da. MS/MS from 551.8 (2+)

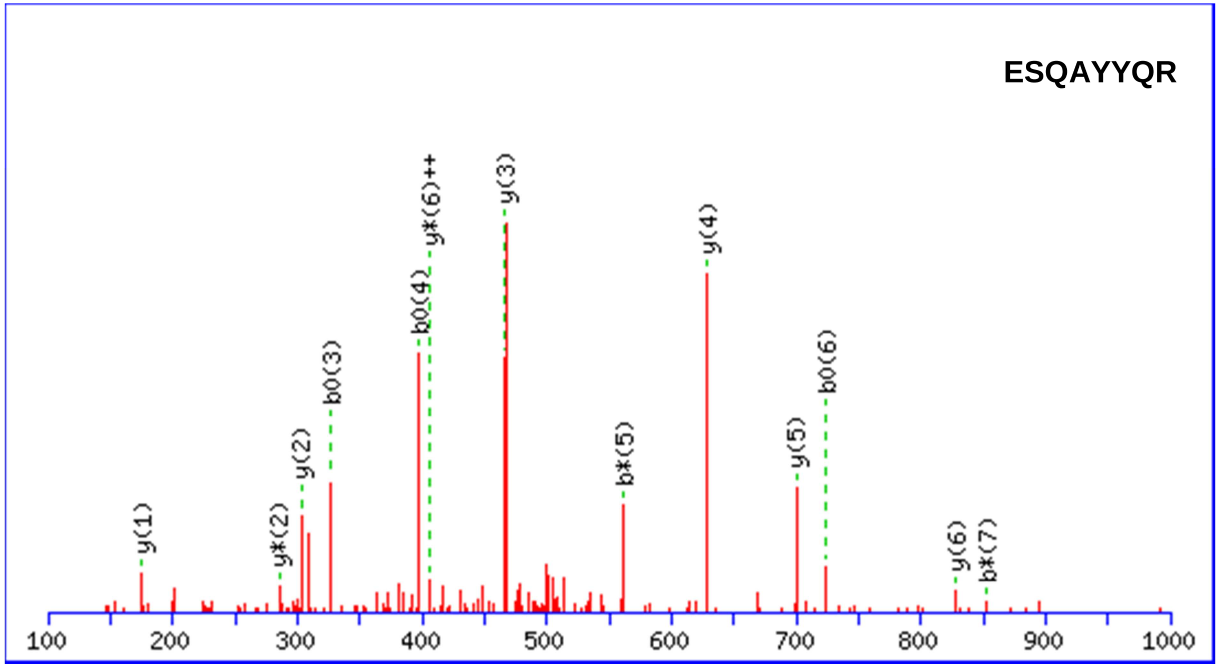

M=1043.5 Da. MS/MS from 522.7 (2+)

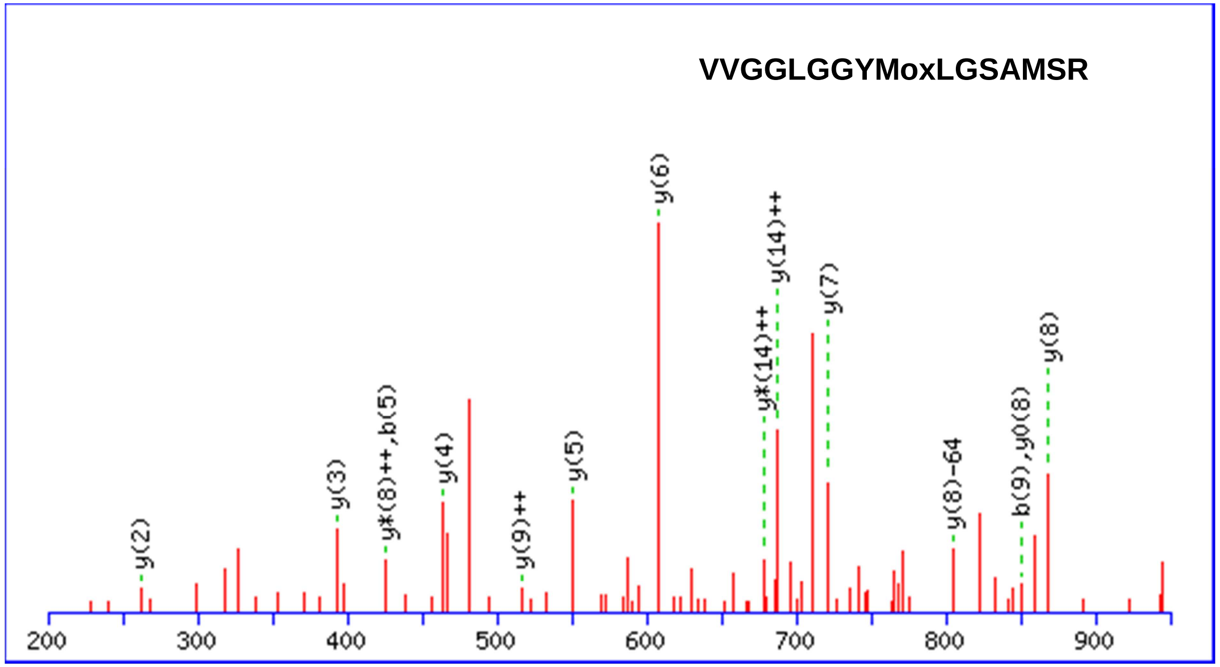

M=1569.7 Da. MS/MS from 785.8 (2+)
